# Pummelo Protects Doxorubicin-Induced Cardiac Cell Death by Reducing Oxidative Stress, Modifying Glutathione Transferase Expression, and Preventing Cellular Senescence

**DOI:** 10.1155/2013/254835

**Published:** 2013-01-21

**Authors:** L. Chularojmontri, O. Gerdprasert, S. K. Wattanapitayakul

**Affiliations:** ^1^Department of Preclinical Sciences, Faculty of Medicine, Thammasat University, Pathum Thani 12120, Thailand; ^2^Department of Anatomy, Faculty of Medicine, Srinakharinwirot University, Bangkok 10110, Thailand; ^3^Department of Pharmacology, Faculty of Medicine, Srinakharinwirot University, Bangkok 10110, Thailand

## Abstract

Citrus flavonoids have been shown to reduce cardiovascular disease (CVD) risks prominently due to their antioxidant effects. Here we investigated the protective effect of pummelo (*Citrus maxima*, CM) fruit juice in rat cardiac H9c2 cells against doxorubicin (DOX-) induced cytotoxicity. Four antioxidant compositions (ascorbic acid, hesperidin, naringin, and gallic acid) were determined by HPLC. CM significantly increased cardiac cell survival from DOX toxicity as evaluated by MTT assay. Reduction of cellular oxidative stress was monitored by the formation of DCF fluorescent product and total glutathione (GSH) levels. The changes in glutathione-S-transferase (GST) activity and expression were determined by enzyme activity assay and Western blot analysis, respectively. Influence of CM on senescence-associated **β**-galactosidase activity (SA-**β**-gal) was also determined. The mechanisms of cytoprotection involved reduction of intracellular oxidative stress, maintaining GSH availability, and enhanced GST enzyme activity and expression. DOX-induced cellular senescence was also attenuated by long-term CM treatment. Thus, CM fruit juice can be promoted as functional fruit to protect cells from oxidative cell death, enhance the phase II GSTP enzyme activity, and decrease senescence phenotype population induced by cardiotoxic agent such as DOX.

## 1. Introduction

Structurally belonging to anthracyclines, doxorubicin (DOX) is an anticancer drug widely used to treat many types of cancer but the dose-dependent cardiotoxic adverse effect limits its full clinical value [1]. It is well recognized that DOX-induced cardiotoxicity occurs through multiple mechanisms which involve oxidative stress generated by quinone moiety of the anthracycline structure. The redox recycling of semiquinone and its parent quinone is known to generate reactive oxygen species (ROS) leading to mitochondria dysfunction, myocyte senescence, and apoptosis, and ultimately causing cardiac remodeling and contractility impairment [2, 3]. Cardiac senescence is associated with the long-term effect of DOX where the clinical manifestration of heart failure may appear several years after DOX administration. Additionally, DOX induced oxidative stress in cardiac myocytes H9c2 represented senescence phenotype similar to characteristics of cardiac cells observed in aging rat [3]. 

Among several attempts initiated to decrease cardiotoxic adverse effect of this valuable drug, scavenging of ROS by natural antioxidants demonstrates favorable cardioprotective effect against DOX-induced cardiotoxicity both *in vitro* and *in vivo* [4, 5]. In many clinical studies related to natural antioxidants, citrus flavonoids and other constituents show prominent effects in reducing cardiovascular disease (CVD) risks [6]. Belonging to the Citrus family, pummelo fruits are indigenous to the oriental areas such as Thailand, China, Japan, and India. Thai pummelo fruit juices contain high antioxidants and scavenging property against free radicals [7], but their potential properties as cytoprotective nutrients against oxidative cell death, particularly DOX toxicity, has not been explored.

In addition to abrogation of oxidative stress by chemically active antioxidants, the removal of anthracycline toxic metabolites by phase II metabolizing enzyme glutathione transferases (previously glutathione-S transferases, GST) has been implicated in the protection of doxorubicin-induced cardiac cell death [8, 9]. Our previous study revealed that GST-Pi (GSTP) is the predominant GST subtype found in H9c2 and played a significant role in nuclear protection against DOX toxicity [9]. In this study, we aimed to investigate the cytoprotective effect of a natural product, pummelo fruit variety “Kao-Tang-Kwa”, on DOX-induced cardiotoxicity in cultured rat cardiomyocyte H9c2 with focus on the modifications of cellular redox stage, GST activity and expression, and cardiac senescence.

## 2. Materials and Methods

### 2.1. Chemicals and Reagents

All chemicals used in this study were analytical or cell culture grade. Internal standards for HPLC analyses of ascorbic acid, hesperidin, naringin, and gallic acid were procured from Sigma-Aldrich (St. Louise, USA). Similarly, doxorubicin and assay reagents for crystal violet cell viability, total GSH levels, and GST activity, ROS determination, and senescence-associated *β*-galactosidase activity (SA-*β*-gal) assay were acquired from Sigma-Aldrich. Cell culture medium, fetal bovine serum, and supplements were supplied by Invitrogen, USA. Oligonucleotides were synthesized by Invitrogen, USA. Reagents for Western blot analysis were purchased from the sources indicated in the specific sections below.

### 2.2. Pummelo Fruit Extract

There are several commercially important varieties of pummelo (*Citrus maxima* (Burm.f.) Merr., CM), fruits in Thailand, including Kao-Tang-Kwa (cucumber-liked white), Kao-Nam-Peung (honey-liked white), Tub-Tim-Siam (Thai ruby), and so forth, of which names describe the characteristics of the inner flesh of the fruit. Each variety belongs to area-specific traditional communities across Thailand while the variety “Kao-Tang-Kwa” is indigenous to Chai-Nat province, Thailand. In this study, the pummelo fruits were harvested from a designated farm in Chai-Nat province and botanically identified by Assoc. Prof. Dr. Ampaiwan Paradornuwat, Faculty of Agriculture, Kasetsart University. The fruit juice was isolated by a fruit extractor and filtered through Whatman No. 1 filter membrane. The filtrate was then prepared in freeze-dried power yielded 8.9% (w/v) and the powder was kept at 4°C until further uses. The aqueous stock solutions of CM (10 mg/mL) were freshly prepared before use.

### 2.3. Analyses of Ascorbic Acid and Citrus Flavonoids Contents Using HPLC

Ascorbic acid content and 3 flavonoids commonly found in the citrus fruits including hesperidin, naringin, and gallic acid were analyzed by reverse-phase HPLC system (Thermo Separation Spectra System P4000) using Luna C18 column (5 *μ*m, 150 × 4.6 mm; Fortune Scientific CO., LTD, Bangkok, Thailand). Standard curves of each reference standard were generated from a series of dilutions 0, 2, 4, 8, 10 *μ*g/mL. The following systems (mobile phase, flow rate, detection wavelength) were applied for the determination of ascorbic acid (100 mM phosphate buffer pH 2.5 and methanol (95 : 5), 0.4 mL/min, 243 nm), hesperidin and naringin (12 mmol heptafluorobutyric acid in 0.05% formic acid and acetronitrile (80 : 20), 1.2, 283 nm), and gallic acid (0.17 M sodium dihydrogen phosphate and methanol (76 : 24), 252 nm) [10–12]. 

### 2.4. H9c2 Cell Culture

The cardiac cell line H9c2 derived from embryonic rat heart was acquired from The American Type Culture Collection (ATCC, CRL-1446). Cells were cultured in Dulbecco's modified Eagle's medium (DMEM), supplemented with antibiotics/antimycotics and 10% fetal bovine serum (FBS) in a humidified atmosphere of 95% air and 5% CO_2_ at 37°C. Culture was replaced with fresh media every 2-3 days and expanded to new culturewares when reached 80% confluency. 

### 2.5. Cell Viability Assay

CM was added to the cell culture 30 min prior to the addition of DOX. H9c2 cells were treated with physiological relevant DOX concentration (0.1 *μ*M) for 48 h with or without coincubation with CM at three different concentrations (10, 100, and 1000 *μ*g/mL). Cell survival was evaluated using crystal violet nuclear staining assay as previously described [13]. Briefly, cells were washed with PBS, and fixed with 10% buffered formalin. Crystal violet solution (0.1% in water/MeOH, 1 : 1) was used to stain nucleus of live cells. Cells were then lysed with 50 mM sodium citrate solution in water/EtOH (1 : 1) and the percentage of cell survival relative to vehicle treatment was quantified by reading the absorbance at 595 nm.

### 2.6. ROS Levels

Determination of intracellular ROS levels were performed by measuring a fluorescent product formed by the oxidation of 2′,7′-dichlorodihydrofluorescein diacetate (DCFHDA, Sigma) and the intracellular ROS. Briefly, the culture media were removed and cells were washed with PBS. Following the addition of fresh culture media, cells were incubated with DCFHDA at the final concentration of 50 *μ*g/mL for 15 min at 37°C. Cells were then wash again with PBS 3 times and the relative amount of fluorescent product was monitored by a microplate reader (Synergy HT, Biotek, USA) with excitation and emission at 485 nm and 528 nm, respectively.

### 2.7. Cellular Glutathione Levels

Reduced glutathione (GSH) is the major antioxidant defense tool both in scavenging actitivity agains ROS and in detoxification of drugs and xenobiotics. The free thiol group provides reducing equivalents for the glutathione peroxidase (GPx) to catalyze reduction of hydrogen peroxide resulted in oxidized glutathione (GSSG) and water. The GSSG-GSH recycle process is then introduced by glutathione reductase (GR) and NADPH. In the process of xenobiotic detoxification, glutathione-S transferase (GST) catalyzes conjugation reaction of GSH to electrophilic substrates such as DOX through the thiol group of GSH. Thus, the availability of GSH pool is crucial for antioxidant defense in biological system.

To assess total cellular GSH (tGSH), the assay was performed according to the method described previously with some modifications [14]. The GSH in cell lysate samples were determined by the conjugation reaction with 5,5′-dithiobis-2-nitrobenzoic acid (DTNB) in assay buffer (100 mM phosphate buffer, 1 mM EDTA, pH 7.4) in the present of GR (1 Unit/*μ*L) and the reaction mixture was incubated at room temperature for 5 min. Then, NADPH (0.3 mg/mL) 50 *μ*L was added. The formation of color product (2TNB) was then monitored at 412 nm for 3 min using kinetic mode (Synergy HT, Biotek, USA). The amounts of total GSH (tGSH) in cell lysates were calculated from GSH standard curves and normalized to 1 mg protein.

### 2.8. GST Activity

The total GST activity was measured in H9c2 using total cell lysates as previously described with minor modifications [9]. Briefly, cultured cells (approximately 1 × 10^7^ cells) were collected in assay buffer (100 mM potassium phosphate buffer with 1.0 mM EDTA and 0.1% Triton X-100, pH 6.5) using cell scraper and allowed to sit on ice for 10 min followed by centrifugation at 2000 ×g for 10 min at 4°C. The supernatants were collected and assayed for protein content using BioRad protein assay kit (BioRad, USA). GST activity was measured in the presence of 0.1 mM GSH and 0.1 mM 1-chloro-2,4-dinitrobenzene (CDNB) in assay buffer. The assay utilized CDNB as substrate for GST isozymes to form conjugated product with the thiol group of glutathione. GST enzyme solutions (0.075 to 0.15 unit/mL) were used as reference for GST activity. The rate of GS-CDNB conjugate formation was monitored for 4 min at 340 nm and GST activity was calculated as follows:

(1) GST  activity  (units/mg  protein)  =ΔA340/min⁡Sample−ΔA340/min⁡BlankProtein  content  (mg/mL).

### 2.9. Western Blot Analysis of GSTP

Since GSTP is the only GST subtype expressed in H9c2 as detected by immunobloting, the effects of CM on the changes in GSTP protein expression of H9c2 were evaluated at 48 h after cells were treated with DOX. Cells were harvested using lysis buffer (20 mM Tris-HCl (pH 7.2), 130 mM NaCl, and 1% NP-40 containing 1% protease inhibitor cocktail (Sigma-Aldrich, P8340)). Cell lysates were normalized for protein content using a Bradford protein assay kit (BioRad, USA). Protein samples were separated by 7.5% SDS-PAGE under reducing conditions and then transferred to a PVDF membrane. The membrane was blocked with 5% nonfat dry milk in TBS (10 mM Tris-HCl (pH 7.5) and 150 mM NaCl) and then incubated at 4°C overnight with anti-GSTP or anti-beta-actin antibody (Santa Cruz Laboratories, USA) in TBS containing 0.1% Tween 20. The blots were washed and then incubated with the peroxidase-conjugated secondary antibodies for 1 h at room temperature. Following several washes, the membrane was developed using the ECL chemiluminescence detection kit (Amersham Biosciences) according to the manufacturer's instructions. The relative expression or immunological reaction bands on the membrane were quantified by band density using beta-actin bands as reference ratio expression.

### 2.10. Senescence-Associated *β*-Galactosidase Activity (SA-*β*-gal) Assay

H9c2 were preincubated with CM for 7 days by replacing the media every 2-3 days with CM-containing media to final concentration of 10, 100, and 1000 *μ*g/mL. Forty-eight hours before the assay, DOX (0.1 *μ*M) was added to the culture media to induce cellular senescence. Beta-galactosidase activity was evaluated as previously described [15]. Briefly, H9c2 cells were washed with PBS and fixed with 2% formaldehyde/0.2% glutaraldehyde for 5 min at room temperature. Following PBS washes, fixed cells were incubated with fresh SA-*β*-gal stain solution (1 mg/mL 5-bromo-4-chloro-3-indyl *β*-D-galactopyranoside (X-gal), 5 mM potassium ferrocyanide, 5 mM potassium ferricyanide, 150 mM NaCl, 2 mM MgCl_2_, 0.01% sodium deoxycholate) for 12–14 h. The development of blue color X-gal product was observed under an inverted microscope. The proportion of cells with blue staining was calculated from total cell counts for at least 300 cells.

### 2.11. Statistical Analysis

Data are presented as the mean ± SEM for at least three independent experiments. Statistical analysis was performed using one-way or two-way ANOVA with Bonferroni's Multiple Comparison Test. A value of *P* < 0.05 was considered statistical significance.

## 3. Results

### 3.1. Ascorbic Acid and Certain Flavonoids Contents in CM

HPLC analysis revealed that CM contained (% w/w) 0.52% ascorbic acid, 0.26% naringin, 0.039% gallic acid while hesperidin was not detectable. When converted to the amounts in 1 Liter CM fruit juice composed of 462 mg ascorbic acid, 231 mg naringin, and 34.6 mg gallic acid. For the purpose of further comparison, the aqueous solutions of CM at 1 mg/mL is corresponding to 29.53 microM ascorbic acid, 2.29 microM gallic acid, and 4.48 microM naringin.

### 3.2. CM Increased Cell Survival in DOX-Induced Cytotoxicity

**Figure 1:**
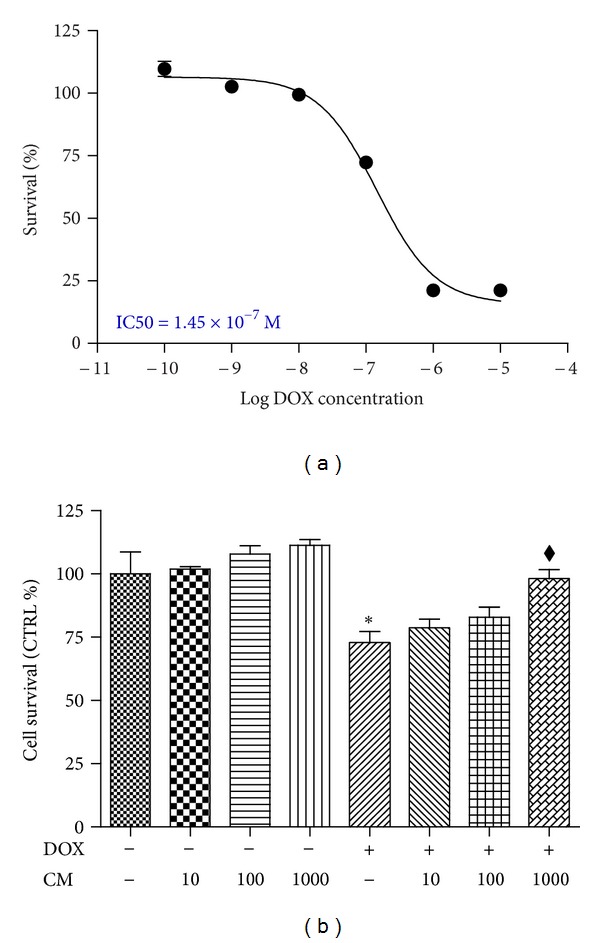
Toxicity of DOX and cytoprotective effect of CM. (a) Dose-response curve of doxorubicin (DOX). H9c2 cells were treated with DOX (0-10-5 M) for 48 h and dose-response curve was obtained with IC50 of 1.45 × 10^−7^ M; (b) cytoprotection of pummelo in DOX-induced cytotoxicity. Cells were incubated for 48 h with DOX (0.1 *μ*M) with or without preincubation with CM at concentrations 10, 100, and 1000 *μ*g/mL. Cell viability was evaluated by crystal violet assay as described in material and Methods. *P < 0.05 versus vehicle treated cells (CTRL); ^*◆*^ *P* < 0.05 versus DOX.

Dose-response curve of DOX cytotoxicity was generated by the 48 h incubation of DOX at a range of concentrations between 10^−10^ to 10^−5^ M and the IC50 was obtained at 1.45 × 10^−7^ M (Figure 1(a)). The concentration of DOX inducing cytotoxicity was selected at 0.1 *μ*M at which it significantly reduced cell survival to 72.74 ± 4.50%. As demonstrated in Figure 1(b), incubation of CM alone (10, 100, and 1000 *μ*g/mL) did not significantly alter cardiac cell survival while cytoprotective effect of CM was observed only at 1000 *μ*g/mL. Cell survival was increased to 96.85 ± 3.15%. In separate experiments, three pure antioxidant compounds detected in CM, including ascorbic acid, naringin, and gallic acid, were tested for cytoprotective effect at the concentrations in the range of 0.1 to 100 *μ*g/mL. H9c2 cell viability was not changed when each compound was incubated with the cardiac cells for 48 h. Coincubation of DOX and each pure antioxidant did not change cell survival at all concentrations of antioxidant used in the experiment (data not shown).

### 3.3. CM Attenuated Cellular Oxidative Stress in DOX-Treated Cells

**Figure 2:**
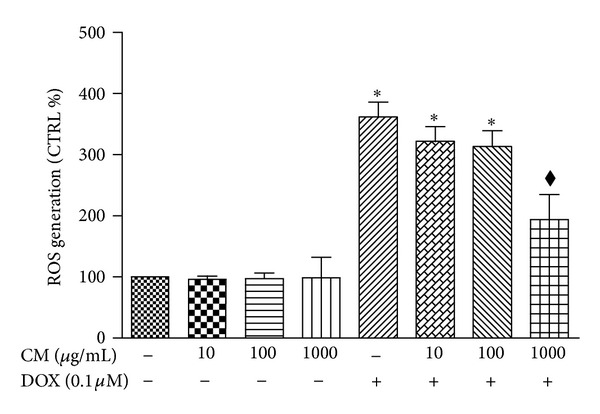
Effect of pummelo on intracellular ROS levels. Cardiac H9c2 cells were treated with DOX (0.1 *μ*M) with or without coincubation with CM at concentrations 10, 100, or 1000 *μ*g/mL for 48 h. Fluorescence intensity of DCF was measured and corresponding to intracellular ROS generation. Data are present as % vehicle treated cells (CTRL). *P < 0.05 versus CTRL; ^*◆*^ *P* < 0.05 versus DOX.

Escalation of ROS level is commonly observed in cells undergone oxidative stress. H9c2 cells treated with DOX (0.1 *μ*M) alone for 48 h showed more than 3-fold increase of intracellular accumulation of ROS while cells receiving CM (10, 100, or 1000 *μ*g/mL) alone did not show significant alteration in ROS levels (Figure 2). Coincubation of CM at 10 and 100 *μ*g/mL did not protect cardiac cells from oxidative stress induced by DOX. However, CM at high concentration (1000 *μ*g/mL) showed significant reduction of intracellular ROS generation although it did not decrease ROS down to the level that comparable to vehicle treated cells. 

### 3.4. Cellular tGSH Pool

**Figure 3:**
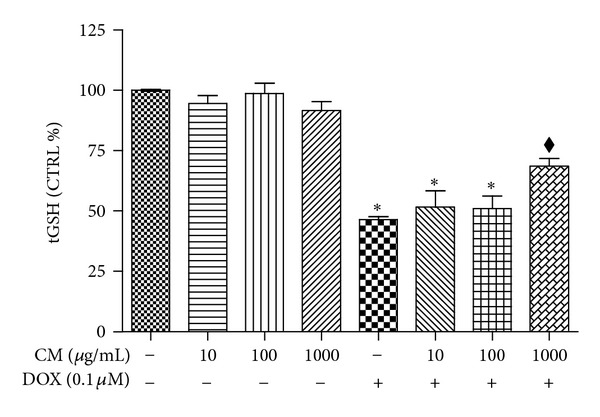
Effect of pummelo on total GSH levels. H9c2 cells were treated with doxorubicin (DOX, 0.1 *μ*M) with or without coincubation with pummelo (CM) at three concentrations as indicated in the figure (10, 100, 1000 *μ*g/mL). The total GSH (tGSH) levels were calculated as described in Materials and Methods. *P < 0.05 versus vehicle treated cells (CTRL); ^*◆*^ *P* < 0.05 versus DOX.

It has been shown that reduction of glutathione pool impairs the cellular capacity in antioxidant defense system and likewise, increased GSH pool is associated with cytoprotection against oxidative damage. In this study as shown in Figure 3, using relative low concentration of DOX at 0.1 *μ*M reduced GSH levels in cardiac cells approximately 17%. Treatment of cells with CM alone at all concentrations studied did not significantly influence GSH levels when compared to those of vehicle treated cells. Coincubation of CM at lower concentrations (10 or 100 *μ*g/mL) with DOX did change GSH antioxidant pool but CM at high concentration (1000 *μ*g/mL) significantly elevated cellular tGSH in DOX-treated cells.

### 3.5. CM Enhanced GST Activity and Expression in H9c2

**Figure 4:**
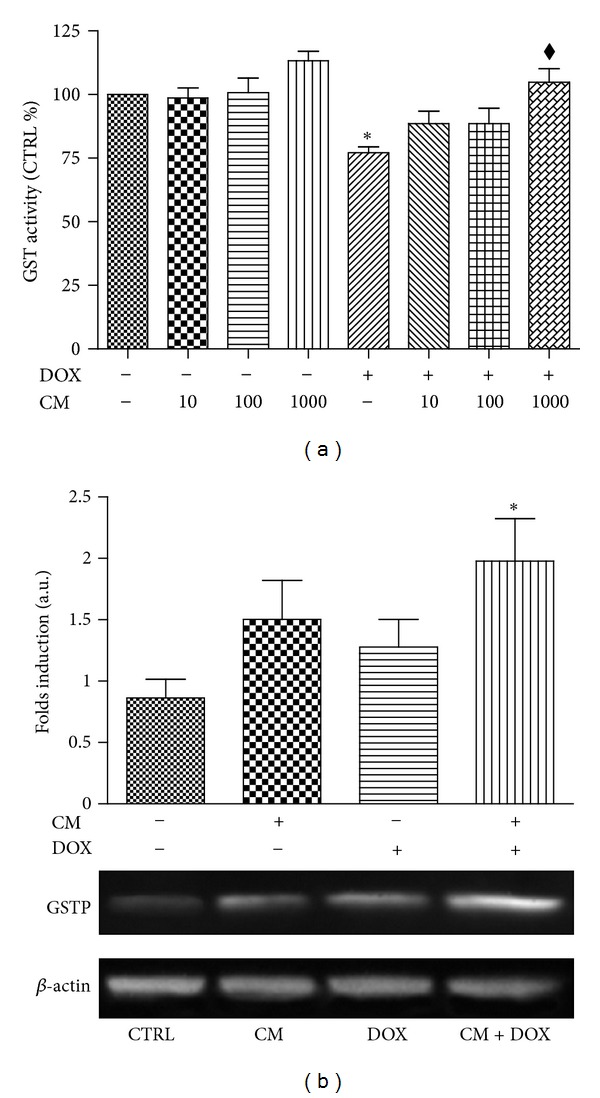
Influence of CM on GST activity and expression in H9c2 cells. Cells were treated with DOX (0.1 *μ*M) and/or pummelo (CM) at concentrations indicated in the Figure. (a) GST activity measurements were performed using total cell lysate as described in Materials and Methods. (b) Western blot analysis of GSTP expression in H9c2 treated with vehicle (CTRL), CM, DOX, or CM and DOX (CM + DOX) for 48 h. *P < 0.05 versus vehicle treated cells (CTRL); ^*◆*^ *P* < 0.05 versus DOX.

Shown in Figure 4 is the GST activity and gene expression in cardiac H9c2 cells. DOX (0.1 *μ*M) caused impairment in GST function approximately one-fourth of those observed in the vehicle treated cardiac cells. CM at 1000 *μ*g/mL significantly improved GST activity to the level comparable to control group while lower concentrations of CM (10 and 100 *μ*g/mL) did not significantly modify GST activity (Figure 4(a)). Western blot analysis revealed that GST protein expression was significantly enhanced in cardiac cells treated with both DOX and CM (1000 *μ*g/mL) which is consistent with GST activity observed in this group. H9c2 cells treated with DOX or CM at 1000 *μ*g/mL alone did not show significant alteration in GST expression (Figure 4(b)).

### 3.6. CM Attenuated Oxidative Stress-Induced Cellular Senescence

**Figure 5:**
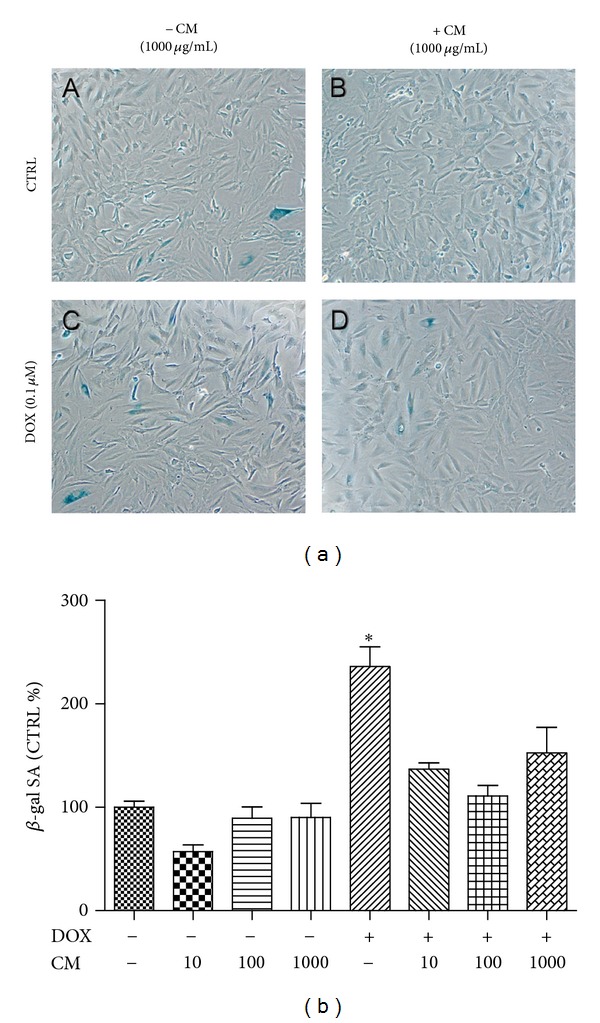
(a) *β*-gal staining in H9c2. Cells were preincubated with pummelo (CM) and/or DOX (0.1 *μ*M) as indicated in the figure and photographed at 100x. (b) Positive cells for *β*-gal SA were counted and calculated as % vehicle treated cells (% CTRL). *P < 0.05 versus CTRL.

In immortalized cell line cultured in growth medium supplemented with growth factors cellular senescence occurs at a very low level. This study used 1% FBS in culture media to sensitize cells to undergo senescence during incubation with DOX. CM alone did not change the proportion of senescence cells in H9c2 culture but an approximately 2-fold increase in *β*-gal-SA was observed in DOX treated cells. CM at all concentrations (10, 100, or 1000 *μ*g/mL) tested in this study significantly mitigated the effect of DOX-induced senescence phenotype in H9c2 (Figure 5).

## 4. Discussion

ROS play an essential role in the development of cardiovascular disease associated with DOX treatment. Our study demonstrated that pummelo fruit juice (*Citrus maxima* (Burm.f.) Merr., CM) protected against DOX-induced cardiotoxicity in H9c2 via mechanisms related to the reduction of cellular oxidative stress, enhancement of GSH antioxidant pool, and increase of the detoxifying enzyme GSTP activity and expression. In addition, long-term pretreatment with CM attenuated DOX-induced cellular senescence in H9c2 cardiac cells.

It is now well recognized that increased ROS generation is the pivotal point upstream of the mechanisms associated with DOX-induced cardiotoxicity [16, 17]. Mitochondria are the primary target and the major source of ROS generation that leads to dysregulation of oxidative metabolism for ATP production [18]. However, strategy to reduce cardiotoxicity from DOX cannot rely upon reduction of ROS level alone. For example, N-acetyly cysteine (NAC) inhibited ROS formation, lipid peroxidation, and restored antioxidant enzyme activities but had modest effect on the protection of DOX-induced cardiac cell death as compared to other natural sources of antioxidants [13, 19]. It imples that alternative mechanisms for attenuation of DOX toxicity such as an increased elimination of DOX by the modification of the phase II detoxification enzyme GSTs may play significant role in cytoprotection [8, 9, 20].

GSTs protect cellular damage against electrophiles and products of oxidative stress, particularly anticancer agents, insecticides, herbicides, and carcinogens. There are two distinct GST superfamilies, microsomal and cytosolic GSTs. While the former involves in endogenous metabolism of leukotrienes and prostaglandins, the latter is a major cytosolic enzyme in some tissues and functions as important detoxification enzyme through GSH-dependant nucleophilic substitution, epoxide ring opening, conjugate addition, ester thiolysis, and so forth. The cytosolic GST superfamily consists of 6 subclasses, including Alpha (GSTA), Mu (GSTM), Omega (GSTO), Pi (GSTP), Theta (GSTT), and Zeta (GSTZ) [21]. GSTs can also be found in the nucleus and in membranes of the endoplasmic reticulum. The degrees of expression can be varied among tissue types and gender differences which may imply tissue's ability to manage specific forms of stress [22]. In this study, pummelo exerted its cytoprotective role in DOX toxicity by increased activity and expression of GSTP in addition to the reduction of ROS stress and maintenance of cellular GSH level. The GST activity in this cardiac cell H9c2 is specific to GSTP subtype since our previous findings indicate that only GSTP protein expression was confirmed by immunoblotting despite mRNA expression of other GST subtypes were detected [9]. CM may partly increase cell survival from DOX toxicity via enhanced elimination of DOX using increased available GST altogether with providing its cofactor GSH. DOX alone did not change GSTP protein expression but its enzyme activity was significantly reduced possibly due to GSH depletion which is similar to cardiac tissue of rats injected with DOX [23]. It is evident that most cellular damage occurs after the depletion of GSH which sets out the onset of uncontrolled oxidative injury. For DOX detoxification, GSTP is an important cytoprotective mechanism as shown in the study that MCF-7 attributed with knockdown GSTP expression enhanced DOX-induced apoptosis [24]. Moreover, study in human lymphocytes revealed that among the enzymes involve in detoxifying gentoxicants including GSTM1, GSTT1, and GSTP1, only GSTP associates with protection against DNA damage specifically induced by DOX [25]. The significance of GSTP is extended beyond conjugation with genotoxic substances, its cytoprotective effect relates to preventive DOX accumulation through forming macromolecular complexes and disruption of JNK-medicated apoptosis pathway [24, 26]. Thus, modification of GSTP may play a crucial role in cardioprotection against DOX.

Despite cardiotoxic effect of DOX having been recognized since early 1970s, its diverse toxic consequences on the heart, acute or late onset, are still not fully understood. The cellular oxidative stress and senescence may associate with molecular mechanism of DOX-induced cardiomyopathy in the latent manifestration of toxicity years after DOX treatment [27]. Maejima et al. demonstrated that neonatal cardiac myocytes treated with low dose DOX (0.1 *μ*M) show evidence of senescence-associated-*β*-galactosidase activity similar to myocytes extracted from aged rats while a higher concentration of DOX (1 *μ*M) triggers apoptotic cell death corresponding to acute DOX toxicity [3]. Our study showed that long-term treatment with CM could significantly attenuate senescence phenotype and reduced intracellular ROS level in cardiac cells treated with low dose DOX (0.1 *μ*M). This effect of CM may related to the reduction of telomerase activity via modification of telomere binding factors 1 and 2 (TRF1, TRF2) and dysregulation of cell cycle regulatory proteins such as checkpoint kinase Chk2 and p53-MAPK signaling which lead to chromosome aberration and delayed cell death due to mitotic catastrophe [28].

Clinical and prospective cohort studies indacated that high consumption of vegetables and fruits, especially citrus fruits enriched with antioxidants, lowers cardiovascular disease risks and prevents the development of certain types of cancer [29–31]. The major antioxidant constituents in citrus fruits consist of ascorbic acid, carotenoids, and unique “citrus flavonoids” including hesperidin, neohesperidin, naringin, narirutin, limonin, and so forth. Ascorbic content in CM fruit juice is approximately 3-fold higher than those of premium tangerine juice cultivated in Northern Thailand [32] but comparable to those of oranges grown in Italy [33] and ponkan tangerine cultivated in Brazil [34]. The HPLC analysis did not detect hesperidine in CM (var. Kao-Tang-Kwa) which is corresponding to the previous study that it only presents in one out of seven pummelo cultivars in Thailand [7]. The flavonoid compositions vary from species and cultivars which are characteristic of species as well as determinant factor for their biological effects. Nonetheless, several studies have shown that synergy among various antioxidants in the extract reflects superior antioxidant activity than that of single component alone. For instance, a well-designed study performed by Snyder et al. [35] indicated that consumption of single flavonoid (hesperidine) had lower capacity than a mix of orange flavonoids (hesperidin, naringenin, and luteolin) at the amounts equivalent to fresh-squeezed navel orange juice in lower plasma antioxidant capacity, total phenolics, and reduction of lipoprotein oxidation.

## 5. Conclusion

Pummelo fruit juice is an excellent source of natural vitamin C and other antioxidant flavonoids supplements. Our study is among the first to provide a better insight into the mechanisms of pummelo in protecting against cytotoxic insults that cause oxidative cell death, specifically to the heart. Pummelo increased cardiac cell survival during DOX treatment through two main mechanisms: (1) the reduction of cellular oxidative stress and enhancement of GSH antioxidant capacity and (2) the elimination of the toxic substance from the cells by increasing the detoxifying GSTP enzyme activity. Long-term treatment with CM inhibited DOX-induced cardiac cells entering senescence-like phenotype which may implicate in the late onset of cardiomyopathy following DOX treatment. Thus, consumption of pummelo fruit may protect against DOX-induced oxidative damage associated with pathogenesis of cardiotoxity. Defining the mechanism of different natural antioxidant action on various forms of oxidative stress is crucial for strategic design of antioxidant therapy in cardiovascular disease.
